# Identification of KHS-101 as a Transcription Factor EB Activator to Promote α-Synuclein Degradation

**DOI:** 10.3390/ijms27020905

**Published:** 2026-01-16

**Authors:** Haizhen Zhu, Anqi Ren, Ting Li, Tao Zhou, Ailing Li, Xin Pan, Liang Chen, Jiayi Chen

**Affiliations:** Nanhu Laboratory, State Key Laboratory of Biomedical Analysis (SKLBA, Formerly Known as National Center of Biomedical Analysis, NCBA), Beijing 100039, China; 15151873536@163.com (H.Z.); anqi.ren1223@foxmail.com (A.R.); tingli@xmail.ncba.ac.cn (T.L.); tzhou@ncba.ac.cn (T.Z.); alli@ncba.ac.cn (A.L.); xpan@ncba.ac.cn (X.P.)

**Keywords:** Parkinson’s disease, TFEB, KHS-101, α-synuclein, lysosome degradation, autophagy–lysosome pathway

## Abstract

Neurodegenerative disorders are increasingly linked to a progressive decline in lysosomal function. Activating Transcription Factor EB (TFEB), a master regulator of lysosomal biogenesis and autophagy, has therefore emerged as a promising therapeutic strategy to enhance cellular clearance in these conditions. In this study, we identified KHS-101 as a novel TFEB activator through a high-throughput screen of blood–brain-barrier-permeable small molecules. We demonstrated that KHS-101 promotes TFEB nuclear translocation, enhances lysosomal biogenesis and proteolytic activity, and increases autophagic flux. Furthermore, KHS-101 significantly accelerates the degradation of pathogenic A53T mutant α-synuclein in a cellular model of Parkinson’s disease, suggesting its potential to mitigate α-synuclein-mediated proteotoxicity and hold neuroprotective potential. Our findings identify KHS-101 as a potent TFEB activator and highlight the therapeutic potential of modulating the autophagy-lysosomal pathway for treating Parkinson’s disease and related disorders.

## 1. Introduction

Lysosomes, the key organelles responsible for degrading macromolecules and cellular components during autophagy, are vital for maintaining cellular homeostasis [1,2,3,4]. Neurons are particularly vulnerable to defects in the clearance of autophagic substrates [5,6,7,8]. This vulnerability stems from their unique polarized structure: the immense volume of axonal and dendritic cytoplasm requires efficient long-distance retrograde transport of autophagic vesicles to the perikaryon, where lysosomes are concentrated for final digestion. Consequently, the neuronal lysosomal system is critically important yet easily overwhelmed, leading to the rapid accumulation of undegraded substrates when lysosomal function is compromised.

A growing body of evidence indicates that lysosomal dysfunction is a common pathological hallmark of multiple neurodegenerative disorders [9,10,11,12,13,14]. These disturbances manifest as aberrant autophagic signaling, accumulation of immature autolysosomes, compromised lysosomal hydrolytic capacity, and failure to clear damaged organelles and protein aggregates. The resulting proteostatic collapse renders neurons vulnerable to oxidative stress and ultimately leads to cell death [15,16,17]. Parkinson’s disease (PD), the second most common neurodegenerative disease, is pathologically characterized by the accumulation of α-synuclein aggregates and the loss of dopaminergic neurons [18]. Notably, the clearance of soluble α-synuclein is primarily mediated by the lysosome through chaperone-mediated autophagy (CMA), whereas the pathogenic α-synuclein aggregates evade CMA and instead rely on macroautophagy for clearance [19,20]. Critically, these aggregates are not merely indigestible substrates; they actively impair the autophagic–lysosomal process by inhibiting CMA and disrupting autophagic flux [21,22,23]. The central role of lysosomal impairment in PD pathogenesis is further underscored by mutations in several PD-associated genes that disrupt the autophagy-lysosomal system, thereby promoting protein aggregation [24,25,26]. This dysfunction creates a vicious cycle wherein impaired lysosomal function leads to α-synuclein accumulation, and the accumulated aggregates further compromise the cell’s clearance capacity, accelerating disease progression. Consequently, enhancing the capacity of the autophagy-lysosomal system has emerged as a compelling therapeutic strategy for PD and related synucleinopathies.

Transcription Factor EB (TFEB), a master regulator of lysosomal biogenesis and autophagy, coordinates the expression of genes involved in these processes by binding to the Coordinated Lysosomal Expression and Regulation (CLEAR) network [27,28]. The activation of TFEB is primarily regulated by its nucleo-cytoplasmic shuttling, which culminates in nuclear translocation and the subsequent initiation of target gene transcription. Impaired autophagy-lysosomal functions due to defective TFEB activity have been implicated in the pathogenesis of multiple neurodegenerative diseases [27]. Notably, TFEB is predominantly excluded from the nucleus in dopaminergic neurons of PD patients. Molecular studies have revealed that α-synuclein directly interacts with and sequesters TFEB in the cytoplasm, thereby inhibiting its nuclear translocation. This α-synuclein-mediated cytoplasmic retention of TFEB creates a vicious cycle that sup-presses autophagic–lysosomal function and further impedes α-synuclein clearance [29]. Importantly, genetic or pharmacological activation of TFEB has been shown to mitigate pathology in various neurodegenerative models by promoting the clearance of toxic protein aggregates [30,31,32,33,34,35]. These compelling findings establish TFEB as an attractive therapeutic target.

However, the clinical translation of TFEB-targeting strategies faces significant challenges. Although several small molecules, such as Rapamycin (NCT04200911) and Mycose (NCT04663854), have entered clinical trials, none have yet been approved for clinical use. Translational development is constrained by several factors. First, many potent TFEB activators, such as mTOR inhibitors, have shown limited efficacy in neurological clinical trials, often due to inadequate central nervous system exposure, systemic toxicity, or off-target effects. For instance, the mTOR-inhibitory activity of such compounds carries a risk of immunosuppression [36,37], and notably, an open-label Phase I trial revealed that rapamycin was undetectable in the cerebrospinal fluid of Alzheimer’s patients, underscoring its poor CNS penetration. Second, other activators like PKC agonists exhibit unacceptable safety profiles, such as tumor-promoting activity, which precludes their therapeutic use. Furthermore, natural products with TFEB-activating properties, such as trehalose, often suffer from low potency, requiring impractically high concentrations for clinical application [38]. Therefore, the discovery of novel, brain-penetrant TFEB activators with improved pharmacological properties represents a critical and unmet medical need.

In this study, to directly address the paramount challenge of central nervous system delivery [39], we specifically employed a blood–brain barrier-permeable compound library for our primary screen. This strategy prioritizes the identification of hits with inherent brain access potential from the outset, thereby avoiding the selection of leads that are inactive in the central nervous system in vivo. We performed a high-throughput screen of this library to identify inducers of TFEB nuclear translocation and identified KHS-101 as a novel, potent TFEB activator that effectively promotes TFEB nuclear translocation without compromising cell viability. We further demonstrate that KHS-101 enhances lysosomal function, increases autophagic flux, and significantly reduces the accumulation of pathogenic A53T mutant α-synuclein in a cellular model of PD. Our work not only identifies KHS-101 as a promising lead compound but also validates the therapeutic potential of modulating the autophagic–lysosomal pathway for treating PD and related neurodegenerative disorders.

## 2. Results

### 2.1. Screening Identifies KHS-101 as an Inducer of TFEB Nuclear Translocation

**Figure 1:**
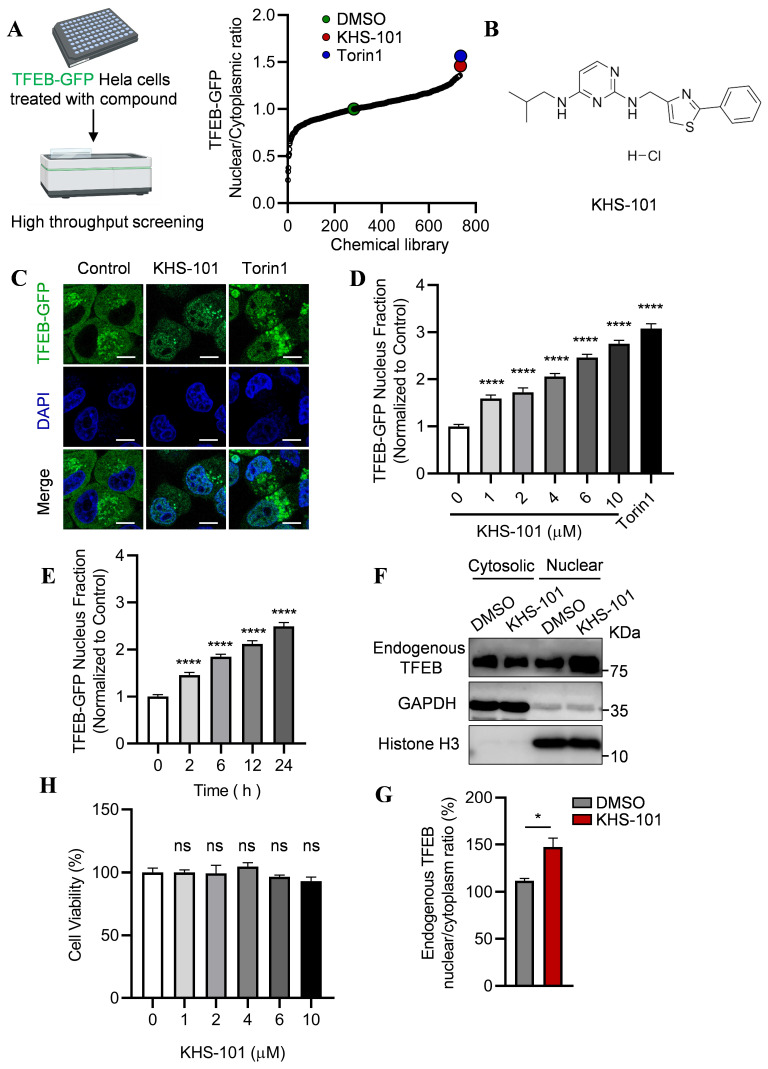
Screening Identifies KHS-101 as an Inducer of TFEB Nuclear Translocation. (**A**) Graphical representation of the chemical screen output. Hela cells stably expressing TFEB-GFP were treated with vehicle (DMSO), library compounds (10 μM) or Torin1 (1 μM) for 24 h. The nuclear/cytoplasmic fluorescence intensity ratio was analyzed. Torin1 served as a positive control. Data are normalized to the vehicle group. (**B**) Chemical structure of KHS-101. (**C**) Representative confocal images showing TFEB-GFP localization. Cells were treated with KHS-101 (10 μM) or Torin1 (1 μM) for 24 h. Scale bars: 10 μm. (**D**,**E**) KHS-101 induces TFEB-GFP nuclear translocation in a dose- and time-dependent manner. Cells were treated with the indicated concentrations of KHS-101 for 24 h (**D**), or with 6 μM KHS-101 for the indicated times (**E**). The nuclear/total fluorescence intensity ratio was analyzed. (**F**,**G**) Representative Immunoblot (**F**) and quantification (**G**) of endogenous TFEB levels in nuclear and cytoplasmic fractions from HeLa cells treated with KHS-101 (6 μM, 6 h). GAPDH and Histone H3 served as loading controls for the cytoplasmic and nuclear fractions, respectively. (**H**) Viability of HeLa cells treated with the indicated doses of KHS-101 for 24 h was not affected. Data are normalized to the control group. All data are expressed as mean ± SEM (* *p* < 0.05; **** *p* < 0.0001, ns means not significant).

Given the critical role of lysosomal function in neurodegenerative diseases, we sought to identify novel, blood–brain-barrier-permeable small molecules that activate TFEB and promote lysosomal biogenesis. To this end, we performed a high-throughput screen using a commercially available library of 734 structurally diverse, bioactive compounds with confirmed blood–brain barrier permeability. The screen was conducted at 10 μM in HeLa cells stably expressing TFEB-GFP, monitoring nuclear translocation of TFEB as the primary readout. From this screen, KHS-101 emerged as a primary hit due to its robust induction of TFEB-GFP nuclear translocation (Figure 1A,B). Torin1, a canonical mTORC1 inhibitor known to induce TFEB nuclear translocation, was used as a positive control [40]. As shown in Figure 1A, Torin1 treatment robustly promoted TFEB-GFP nuclear localization compared to the vehicle control.

**Figure A1:**
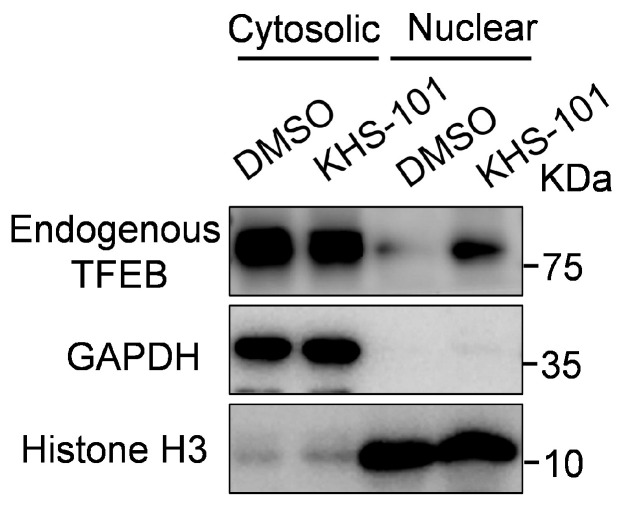
KHS-101 promotes TFEB nuclear translocation in SH-SY5Y cells. Immunoblot of endogenous TFEB levels in nuclear and cytoplasmic fractions from SH-SY5Y cells treated with KHS-101 (6 μM, 6 h). GAPDH and Histone H3 served as loading controls for the cytoplasmic and nuclear fractions, respectively.

To validate and characterize the activity of KHS-101, we treated TFEB-GFP HeLa cells with increasing concentrations of the compound. KHS-101 induced TFEB nuclear translocation at concentrations as low as 1 μM, with a clear dose-dependent effect observed between 1 and 10 μM (Figure 1C,D). Time-course analysis showed that KHS-101 triggered significant nuclear translocation within 2 h, and the effect increased in a time-dependent manner up to 24 h (Figure 1E). To confirm that this effect was not an artifact of TFEB-GFP overexpression, we examined endogenous TFEB localization by cellular fractionation. Immunoblot analysis revealed that KHS-101 potently induced the translocation of endogenous TFEB from the cytosol to the nucleus (Figure 1F,G; Figure A1). Furthermore, KHS-101, at its active concentrations (1–10 μM), did not affect cell viability, confirming that the observed TFEB activation is not a consequence of cytotoxic stress.

**Figure A2:**
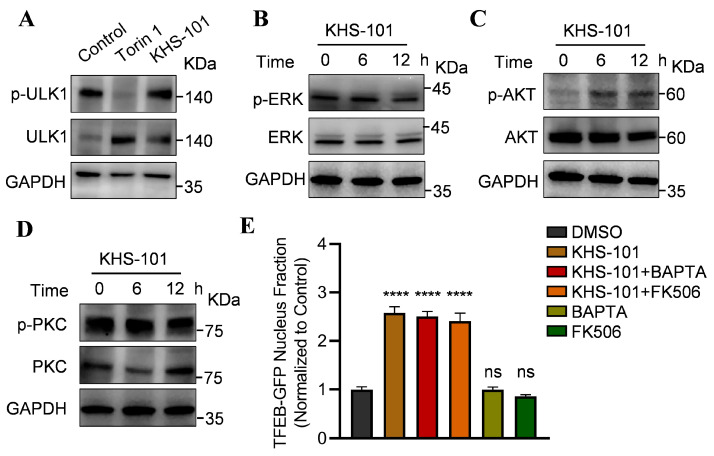
KHS-101 promotes TFEB nuclear translocation independently of mTOR, AKT, ERK, PKC, and calcineurin signaling. (**A**) KHS-101 does not inhibit the mTOR signaling pathway. HeLa cells were treated with KHS-101 (10 μM) for 12 h, and phosphorylation levels of ULK1 were detected by Western blotting. Torin 1 (1 μM) was used as a positive control. (**B**,**C**) KHS-101 does not suppress phosphorylation of ERK or AKT. HeLa cells were treated with KHS-101 (10 μM) for the indicated durations, and levels of p-ERK/ERK and p-AKT/AKT were detected by Western blotting. (**D**) KHS-101 does not enhance PKC phosphorylation. HeLa cells were treated with KHS-101 (10 μM) for the indicated times, and p-PKC/PKC levels were detected by Western blotting. (**E**) KHS-101–induced TFEB nuclear translocation is not inhibited by calcium chelation or calcineurin blockade. TFEB-GFP HeLa cells were pretreated with BAPTA-AM (20 μM) or FK506 (10 μM) for 30 min, followed by co-treatment with KHS-101 for 24 h. Nuclear/total fluorescence intensity ratios were quantified. All data are expressed as mean ± SEM (**** *p* < 0.0001, ns means not significant).

We next investigated whether KHS-101 induces TFEB nuclear translocation through major known regulatory pathways. TFEB subcellular localization is tightly controlled by phosphorylation [27]. mTOR kinase is a major kinase that phosphorylates TFEB to promote its cytosolic retention [41,42,43]. However, KHS-101 treatment for 12 h did not suppress ULK1 phosphorylation, indicating that its mechanism of action is independent of mTORC1 (Figure A2A). By contrast, the mTORC1 inhibitor Torin1 effectively suppressed ULK1 phosphorylation (Figure A2A). We also examined other kinases known to inhibit TFEB nuclear translocation, including AKT and ERK [28,38], and found that KHS-101 did not reduce their phosphorylation, ruling out the involvement of these pathways (Figure A2B,C). In addition, PKC phosphorylates TFEB to promote its nuclear translocation [44]. KHS-101 did not increase PKC activity (Figure A2D). Calcineurin, which is typically activated by elevated intracellular calcium levels, dephosphorylates TFEB to facilitate its nuclear translocation [45,46,47]. However, co-treatment with the calcium chelator BAPTA-AM or the calcineurin inhibitor FK506 failed to attenuate KHS-101-induced TFEB translocation, indicating that the calcium-calcineurin axis is also not essential for its action (Figure A2E). Together, these results demonstrate that KHS-101 activates TFEB through a mechanism distinct from these canonical regulatory pathways.

Collectively, these data identify KHS-101 as a novel and potent activator of TFEB, capable of inducing its nuclear translocation in a dose- and time-dependent manner without compromising cell viability. The activation occurs through a mechanism distinct from known canonical pathways, suggesting that KHS-101 may act through a previously uncharacterized target to promote TFEB activation.

### 2.2. KHS-101 Promotes Lysosomal Function

**Figure 2:**
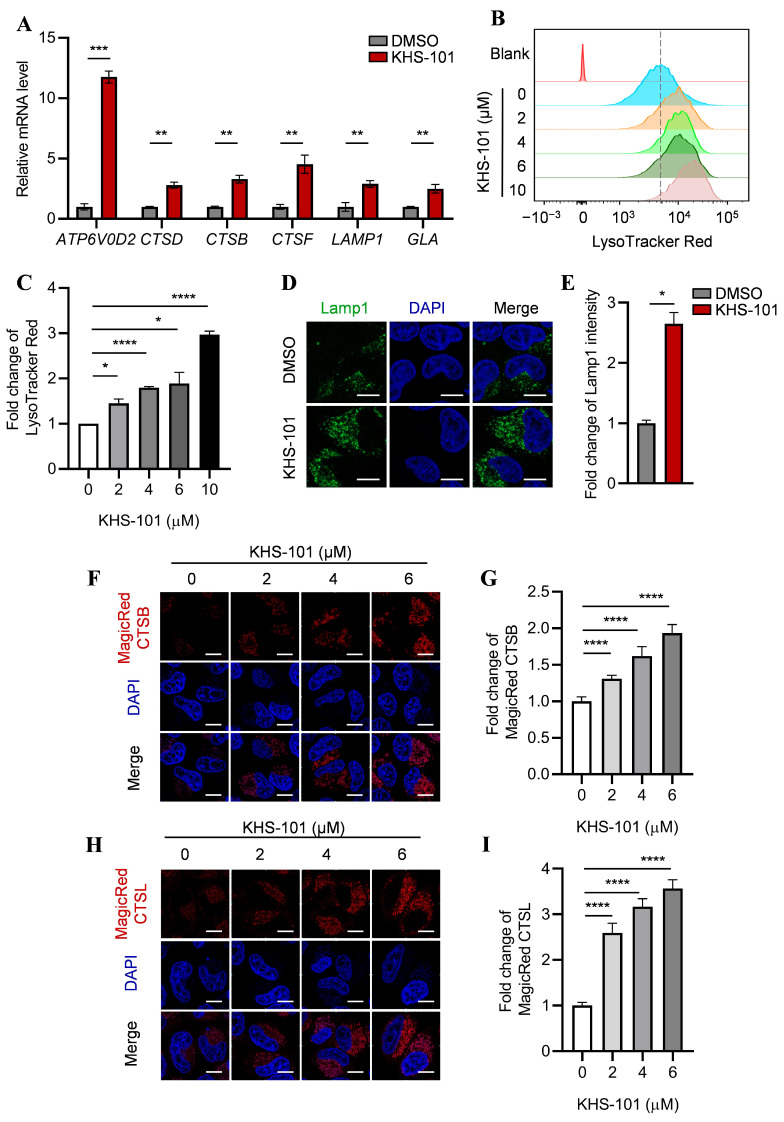
KHS-101 promotes lysosomal function. (**A**) KHS-101 induces the expression of lysosomal genes. Hela cells were treated with KHS-101 (6 μM, 24 h) and subjected to qRT-PCR analysis. (**B**,**C**) KHS-101 increases LysoTracker Red staining. Representative images (**B**) and quantification (**C**) of LysoTracker Red staining in Hela cells treated with KHS-101 (2, 4, 6, 10 μM) for 24 h. The dotted line indicates the peak LysoTracker Red fluorescence intensity of the untreated control (0 μM KHS-101) group. (**D**,**E**) enhances expression of the lysosomal marker LAMP1. Representative images (**D**) and quantification (**E**) of normalized relative fluorescence intensities for Lamp1 in Hela cells treated with 6 μM KHS-101 for 24 h. Scale bars: 10 μm. (**F**–**I**) KHS-101 enhances CTSB and CTSL activity. Representative images (**F**,**H**) and quantification (**G**,**I**) of normalized relative fluorescence intensities for Magic Red CTSB or CTSL in Hela cells treated with KHS-101 (2, 4, 6 μM) for 24 h. Scale bars: 10 μm. All data are expressed as mean ± SEM (* *p* < 0.05; ** *p* < 0.01; *** *p* < 0.001; **** *p* < 0.0001).

Nuclear translocation of TFEB is known to drive the expression of genes coordinating lysosomal biogenesis and function [48]. To determine whether the observed nuclear translocation of TFEB following KHS-101 treatment led to transcriptional activation of its target genes, we performed quantitative real-time PCR in HeLa cells treated with 6 μM KHS-101 for 24 h. The analysis revealed significant upregulation of key TFEB target genes, including those encoding lysosomal membrane proteins (*LAMP1*), lysosomal hydrolases (*CTSB*, *CTSD*, *CTSF*, *GLA*), and a component of the vacuolar ATPase complex (*ATP6V0D2*) (Figure 2A).

**Figure A3:**
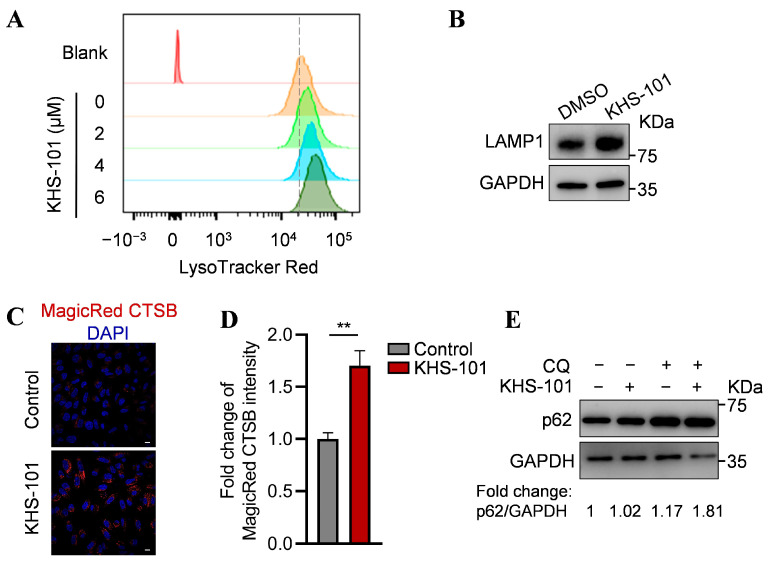
KHS-101 promotes lysosomal function and enhances autophagic flux. (**A**) KHS-101 increases LysoTracker Red staining in SH-SY5Y cells treated with KHS-101 (2, 4, 6 μM) for 24 h. The dotted line indicates the peak LysoTracker Red fluorescence intensity of the untreated control (0 μM KHS-101) group. (**B**) KHS-101 increases the protein level of LAMP1 in Hela cells treated with 6 μM KHS-101 for 24 h. (**C**,**D**) KHS-101 enhances CTSB activity in SH-SY5Y cells. Representative images (**C**) and quantification (**D**) of normalized relative fluorescence intensities for Magic Red CTSB in SH-SY5Y cells treated with 6 μM KHS-101 for 24 h. Scale bars: 10 μm. (**E**) KHS-101 enhances autophagic flux in SH-SY5Y cells. Cells were treated with KHS-101 (6 μM) with or without CQ (20 μM) for 24 h, followed by immunoblot analysis of p62 levels. All data are expressed as mean ± SEM (** *p* < 0.01).

We next assessed the functional consequences of this transcriptional program on lysosomes. Flow cytometric analysis of cells stained with LysoTracker Red, a dye that accumulates in acidic organelles, showed a marked increase in signal upon KHS-101 treatment, indicating an elevated number of acidic lysosomes. (Figure 2B,C; Figure A3A). Consistent with enhanced lysosomal biogenesis, immunofluorescence and Western blot analysis demonstrated a significant increase in the protein level of LAMP1 (Figure 2D,E; Figure A3B). To directly evaluate lysosomal hydrolytic capacity, we measured the activity of cathepsin B and L using fluorogenic substrates (Magic Red CTSB and CTSL), which yield fluorescence upon proteolytic cleavage. KHS-101 treatment induced a dose-dependent increase in fluorescence from both substrates, providing direct evidence that the newly formed lysosomes are functionally active. (Figure 2F–I; Figure A3C,D).

Collectively, these data demonstrate that KHS-101 not only induces the expression of genes critical for lysosomal biogenesis but also promotes the formation of acidic, protease-active lysosomes, confirming a functional enhancement of the lysosomal system.

### 2.3. KHS-101 Enhances Autophagic Flux

**Figure 3:**
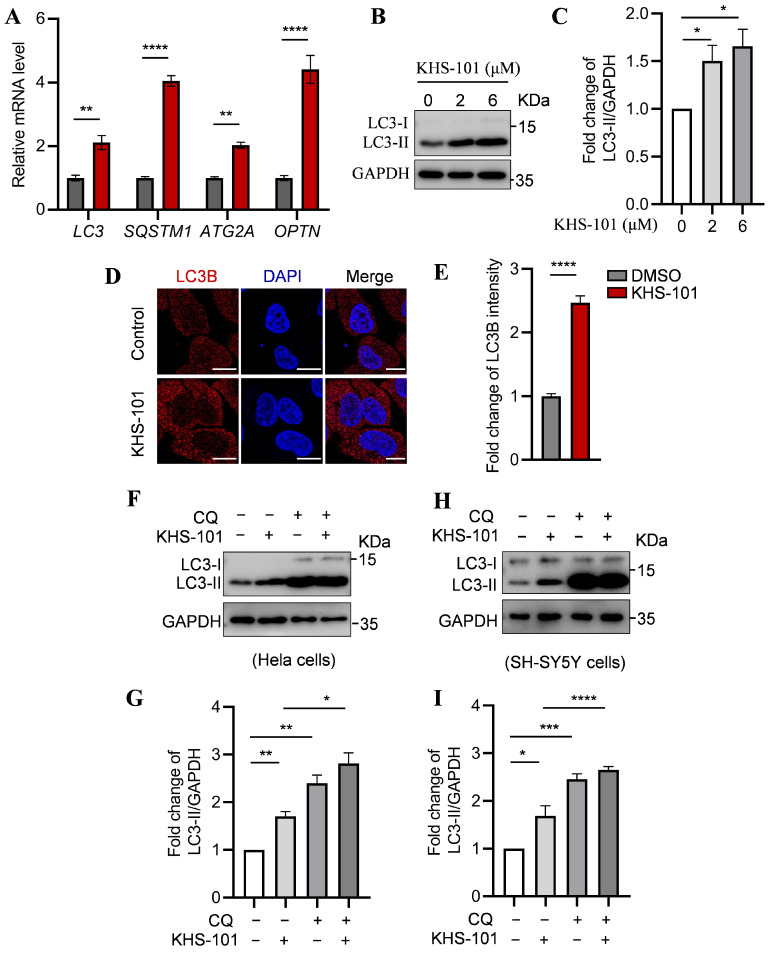
KHS-101 enhances autophagic flux. (**A**) KHS-101 induces the expression of autophagy-related genes. Hela cells were treated with KHS-101 (6 μM, 24 h) and analyzed by qRT-PCR. (**B**–**E**) KHS-101 increases LC3-II accumulation. Representative immunoblots (**B**) and quantification (**C**) of LC3-II levels in HeLa cells treated with KHS-101 (2 μM, 6 μM) for 24 h. Representative immunofluorescence images (**D**) and quantification (**E**) of LC3 puncta in HeLa cells treated with KHS-101 (6 μM, 24 h). Scale bars: 10 μm. (**F**–**I**) KHS-101 enhances autophagic flux in Hela (**F**,**G**) and SH-SY5Y (**H**,**I**) cells. Cells were treated with KHS-101 (6 μM) with or without CQ (40 μM for Hela, 20 μM for SH-SY5Y) for 24 h, followed by immunoblot analysis of LC3-II levels. All data are expressed as mean ± SEM (* *p* < 0.05; ** *p* < 0.01; *** *p* < 0.001; **** *p* < 0.0001).

Given that TFEB is a master regulator of genes involved in multiple stages of autophagy [49], we investigated whether KHS-101-mediated TFEB activation enhances the autophagic process. Quantitative PCR analysis revealed that KHS-101 treatment significantly upregulated the expression of key autophagy-related genes, including those involved in initiation (*ATG2A*), cargo recognition (*OPTN*), autophagosome formation and elongation (*LC3B*), and autophagic degradation (*SQSTM1*) (Figure 3A). Consistent with this transcriptional upregulation, we observed a marked increase in the lipidated form of LC3 (LC3-II), a key marker of autophagosomes [50], by both Western blotting and immunofluorescence microscopy (Figure 3B,C).

To determine whether the accumulation of LC3-II reflected enhanced autophagic flux or a blockade of lysosomal degradation, we treated cells with KHS-101 in the presence of chloroquine (CQ), a lysosomal inhibitor that blocks autophagosome–lysosome fusion and subsequent cargo degradation [51]. In both HeLa and SH-SY5Y cells, co-treatment with KHS-101 and CQ resulted in a greater accumulation of LC3-II compared to KHS-101 alone (Figure 3D,E). Consistently, co-treatment with KHS-101 and CQ leads to a greater accumulation of SQSTM1/p62 compared to either treatment alone (Figure A3E). These data demonstrate that KHS-101 promotes autophagosome formation upstream of lysosomal degradation, confirming a genuine increase in autophagic flux.

### 2.4. KHS-101 Promotes Clearance of Mutant α-Synuclein via the Autophagic-Lysosomal Pathway

**Figure 4:**
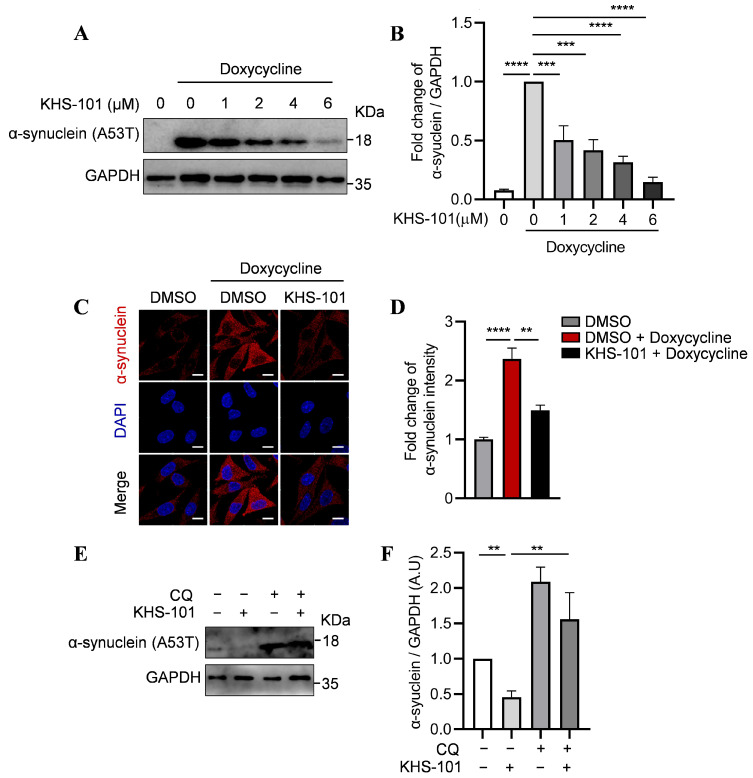
KHS-101 increases α-synuclein (A53T) degradation via autophagic–lysosomal system. (**A**,**B**) Representative immunoblot (**A**) and quantification (**B**) of α-synuclein levels. SH-SY5Y cells were treated with KHS-101 (1 μM, 2 μM, 4 μM, 6 μM) and doxycycline (1 μg/mL) for 48 h. (**C**,**D**) Representative immunofluorescence images (**C**) and quantification (**D**) of α-synuclein levels in SH-SY5Y cells obtained after 48 h treatment with KHS-101 (6 μM) and doxycycline (1 μg/mL). Scale bars represent 10 μm. (**E**,**F**) α-synuclein degradation by KHS-101 was abolished in the presence of CQ. SH-SY5Y cells were treated with KHS-101 (6 μM) and doxycycline (1 μg/mL) for 48 h with or without CQ (5 μM). All data are expressed as mean ± SEM (** *p* < 0.01; *** *p* < 0.001; **** *p* < 0.0001).

Given that TFEB activation mitigates toxic protein accumulation in neurodegenerative diseases such as Parkinson’s disease (PD) [29,30], we investigated whether KHS-101 reduces levels of A53T mutant α-synuclein, a key pathological hallmark of PD. Using a doxycycline-inducible SH-SY5Y cell model expressing A53T mutant α-synuclein, we found that KHS-101 treatment reduced α-synuclein levels in a dose-dependent manner, as shown by Western blot analysis (Figure 4A,B). This reduction was confirmed by immunofluorescence staining (Figure 4C,D).

To determine whether α-synuclein clearance was dependent on the autophagic–lysosomal pathway, we co-treated cells with KHS-101 and the lysosomal inhibitor CQ. The enhancement of α-synuclein degradation by KHS-101 was abolished in the presence of CQ (Figure 4E,F). Collectively, these data demonstrate that KHS-101 facilitates clearance of pathogenic A53T α-synuclein in a manner dependent on a functional autophagic–lysosomal system.

## 3. Discussion

The accumulation of misfolded protein aggregates represents a hallmark of neurodegenerative diseases, driven by the collapse of cellular protein homeostasis [52,53,54,55]. Clearance of pathogenic protein aggregates such as α-synuclein constitutes a central therapeutic goal in PD [56,57]. Strategic activation of TFEB to enhance the autophagic–lysosomal pathway has therefore emerged as a promising therapeutic strategy [27,38,58,59]. Our study identifies KHS-101 as a novel, brain-penetrant small-molecule activator of TFEB that effectively promotes clearance of pathogenic α-synuclein. These findings not only offer a promising therapeutic candidate for PD but also provide direct experimental support for enhancing the autophagic–lysosomal pathway to ameliorate proteostatic impairment.

Current strategies to mitigate α-synuclein pathology in PD include immunotherapies targeting extracellular aggregates [60,61,62,63,64], and antisense oligonucleotides suppressing *SNCA* expression [65,66,67]. Our work aligns with an alternative approach: enhancing the brain’s intrinsic clearance capacity through direct activation of TFEB, the master regulator of lysosomal biogenesis. We demonstrate that KHS-101 facilitates lysosome-dependent clearance of pathogenic A53T α-synuclein without compromising cell viability, indicating a favorable therapeutic window.

The clinical translation of TFEB activators has been limited by the drawbacks of existing agents. Notably, mTORC1 inhibitors like rapamycin, despite their potency, carry risks of immunosuppression and have shown limited brain exposure in clinical settings [36,37]; PKC agonists (e.g., PMA), which are tumor-promoting and therapeutically unsuitable; and natural products such as trehalose, which exhibit weak potency (∼100 mM) despite proposed mechanisms like AKT inhibition [38]. In contrast to these agents, KHS-101 demonstrates a distinct profile: it acts through an mTOR-independent mechanism, suggesting a potentially improved safety margin regarding immune modulation; it induces rapid TFEB nuclear translocation within two hours at low micromolar concentrations, showing significantly greater potency than trehalose; and crucially, it elicits a genuine functional enhancement of the autophagic–lysosomal pathway, as opposed to lysosomal inhibitors like chloroquine that indirectly trigger TFEB translocation via stress signaling [43]. Our high-throughput screen of a blood–brain barrier-permeable compound library successfully identified KHS-101 as a potent TFEB nuclear translocation activator.

TFEB nucleo-cytoplasmic shuttling is classically regulated by phosphorylation–dephosphorylation dynamics, involving kinases such as mTOR, ERK, AKT, and PKC, and phosphatases like calcineurin [27]. Our data show that KHS-101 activates TFEB independently of all these canonical regulatory nodes. This, combined with its rapid onset of action, strongly suggests that KHS-101 may operate through a novel, previously uncharacterized mechanism. We hypothesize that it may either directly interact with TFEB to influence its conformation or subcellular localization, or it may modulate a novel upstream component of the TFEB regulatory network. This distinct mechanism of action not only differentiates KHS-101 from existing tool compounds but also positions it as a unique chemical probe to uncover new biology within the lysosomal regulatory axis.

In conclusion, our work establishes KHS-101 as a potent activator of the TFEB-lysosomal pathway with significant efficacy in a cellular model of PD. While the present study provides robust in vitro proof-of-concept, several critical future directions are warranted. First, target identification efforts (e.g., through chemical proteomics or functional genomics screens) are essential to elucidate its precise molecular mechanism. Second, evaluation in animal models of PD and related synucleinopathies is needed to confirm its in vivo efficacy, pharmacokinetics, and safety profile. To further delineate the mechanistic details of KHS-101 action, future work could also aim to: (i) employ TFEB transcriptional reporter assays; (ii) utilize detailed biophysical measurements to define its effects on lysosomal properties such as intraluminal pH; (iii) determine its specificity for different pathogenic forms of α-synuclein (e.g., soluble oligomers versus insoluble fibrils); and (iv) investigate its effects on the subcellular trafficking and secretion of α-synuclein. Third, investigating its effects on other disease-relevant protein aggregates (e.g., tau, huntingtin) will determine its broader therapeutic potential. Given that proteostasis failure is a common feature of these diseases, KHS-101 represents both a valuable tool for investigating TFEB biology and a promising lead candidate for developing disease-modifying therapies aimed at restoring proteostasis.

## 4. Materials and Methods

### 4.1. Cell Culture

Hela (CCL-2) and SH-SY5Y (CRL-2266) cells were obtained from the American Type Culture Collection (ATCC), Manassas, VA, USA. All cells were cultured in Dulbecco’s modified Eagle’s medium (DMEM) supplemented with 10% fetal bovine serum (FBS), 100 μg/mL streptomycin, and 100 U/mL penicillin. Cells were incubated at 37 °C in a humidified incubator with 5% CO_2_. Cell lines were authenticated by short tandem repeat (STR) profiling and confirmed to be free of mycoplasma contamination by regular PCR-based testing.

### 4.2. Plasmid Construction and Lentiviral Transduction

The cDNA sequences of TFEB and α-synuclein (A53T) were purchased from Sino Biological (Beijing, China). All overexpression cell lines were established via lentiviral transduction. To generate HeLa cells stably expressing TFEB-GFP, the TFEB sequence was cloned into the pCDH-T2B-copGFP vector. Transduced cells were selected by fluorescence-activated cell sorting (FACS) based on GFP expression. For inducible expression of α-synuclein (A53T) in SH-SY5Y cells, the gene was cloned into the pLVX-TetOne-Puro vector. Cells were selected with 8 μg/mL puromycin and induced with 1 μg/mL doxycycline.

### 4.3. High-Content Chemical Screening

A library of blood–brain barrier-permeable compounds (MedChemExpress, Monmouth Junction, NJ, USA) was screened at a final concentration of 10 μM in TFEB-GFP HeLa cells. After 24 h incubation, cells were stained with DAPI (ZsBio, ZLI-9557, Beijing, China) and imaged using a 20× objective on an Operetta automated microscope (PerkinElmer, Waltham, MA, USA) with 488 nm excitation and 509 nm emission. Image analysis was performed using Harmony software (v4.9). The nuclear translocation of TFEB-GFP was quantified by measuring the total fluorescence intensity within the nuclear and cytoplasmic compartments of individual cells. The Nuclear Fraction was calculated as the ratio of total nuclear intensity to total cellular (nuclear + cytoplasmic) intensity, and normalized to the mean value of the vehicle (DMSO) control group on each plate.

### 4.4. Immunofluorescence

Cells grown on coverslips were fixed with 4% paraformaldehyde for 10 min, permeabilized with 0.3% Triton X-100 (Sigma-Aldrich, Darmstadt, Germany), and blocked with 3% goat serum in PBS. Primary antibodies included: mouse anti-LAMP1 (Santa Cruz, Dallas, TX, USA, sc-20011, 1:400), rabbit anti-LC3B (Sigma, Kawasaki, Japan, L7543, 1:400), and rabbit anti-α-synuclein (Proteintech, Rosemont, IL, USA, 10842-1-AP, 1:200). After incubation with secondary antibodies, coverslips were mounted with VectaShield containing DAPI. Images were acquired using a 60× oil immersion lens on a ZEISS LSM 880 microscope (ZEISS, Göttingen, Germany) and analyzed with Volocity 6.1.1.

### 4.5. Magic Red Staining

Cells were stained with Magic Red CTSB or CTSL substrate according to the manufacturer’s instructions (ImmunoChemistry Technologies, Davis, CA, USA, ICT-938 and ICT-942). After 30 min incubation at 37 °C, cells were washed with PBS and imaged on a ZEISS LSM 880 microscope. Fluorescence was quantified using Volocity 6.1.

### 4.6. LysoTracker Red Staining

Lysotracker Red staining was carried out as the manufacturer’s instructions. Cells were stained with Lysotracker Red DND-99 50 nmol/L (Thermo Fisher Scientific, Waltham, MA, USA, L7528) in warmed DMEM medium for 30 min at 37 °C, washed with PBS, and examined by flow cytometry. The data was analyzed using FlowJo v10 software.

### 4.7. Western Blotting

Cells were collected and resuspended in RIPA lysis buffer (50 mM Tris, pH 7.4, 150 mM NaCl, 1% NP-40 (Santa Cruz Biotechnology, Dallas, TX, USA, CAS 9016–45-9), 0.5% Na-deoxycholate (Sigma Aldrich, D6750, St. Louis, MO, USA) containing certain protease inhibitors 0.1 mM phenylmethanesulfonyl fluoride (Target Molecule Corporation, Boston, MA, USA, T0789), 1× EDTA-free Protease Inhibitor Cocktail (Medchemexpress, Monmouth Junction, NJ, USA, HY-K0010) on ice. Antibodies were used at the following concentrations: rabbit anti-TFEB (Proteintech, 13372-1-AP, 1:1000), HRP Conjugated anti-GAPDH (Servicebio, Wuhan, China, ZB15004-HRP-100, 1:5000), rabbit anti-Histone H3 (Cell signaling technology, Danvers, MA, USA, 9715S, 1:1000), rabbit anti-LC3B (Sigma, l7543, 1:5000), rabbit anti-α-synuclein (Proteintech, 10842-1-AP, 1:1000), Phospho-ULK1 (Ser757) (D7O6U) Rabbit Monoclonal Antibody (Cell signaling technology, 14202S, 1:1000), ULK1 (D8H5) Rabbit Monoclonal Antibody (Cell signaling technology, 8054S, 1:1000), Phospho-p44/42 MAPK (Erk1/2) (Thr202/Tyr204) Antibody (Cell signaling technology, 9101S, 1:1000), p44/42 MAPK (Erk1/2) (137F5) Rabbit Monoclonal Antibody (Cell signaling technology, 4695S, 1:1000), Phospho-Akt (Ser473) Antibody (Cell signaling technology, 9271S, 1:500), Akt (pan) (40D4) Mouse Monoclonal Antibody (Cell signaling technology, 2920S, 1:500), Phospho-PKC (pan) (beta II Ser660) Antibody (Cell signaling technology, 9371T; 1:1000), PKC Antibody (A-3) (Santa Cruz Biotechnology, sc-17769, 1:500), mouse anti-LAMP1 (Santa Cruz, sc-20011, 1:500), rabbit anti-p62 (MBL Life Science, Tokyo, Japan, PL045, 1:5000). Membranes were incubated with antibody overnight at 4 °C, and then incubated with HRP-conjugated secondary antibodies (Dako Cytomation, Glostrup, Denmark, P0448; 1:5000) for 1 h at room temperature, immunopositive bands were visualized with Supersignal West Femto chemiluminescent substrate (Pierce, Appleton, WI, USA, 34095).

### 4.8. RNA Isolation and qRT-PCR

Total RNA were extracted using TRIzol Reagent (Invitrogen, Carlsbad, CA, USA, 15596018) and used to prepare cDNA with PrimeScript™ RT Master Mix (TAKARA, Kyoto, Japan, RR036A). Quantification of the indicated gene expression was carried out by quantitative real-time PCR using PowerUp™ SYBRTM Green Master Mix (Thermo Fisher Scientific, A25742). *GAPDH* was used as an internal control, and each sample was run in triplicate; the primers are provided in Table A1.

### 4.9. Cell Viability Assay

The cell toxicity was measured according to the manufacturer’s instructions using CellTiter 96^®^ AQueous Non-radioactive Cell Proliferation Assay (MTS) (Promega, Madison, WI, USA, G5421). Briefly, cells were added 5% MTS diluted in warmed DMEM medium, and incubated for 1 h at 37 °C in a 5% CO_2_ and 95% air humidified incubator. The result data was measured by a microplate reader and subtracted from the values of the blank group, and then we normalized control (0 μM) to ‘100%’.

### 4.10. Subcellular Fractionation

Separation of cytoplasmic and nuclear fractions was carried out according to the manufacturer’s instructions using the cell fraction kit (Cell signaling technology, 9038S). Briefly, cell pellets washed with PBS were resuspended in Cytoplasm Isolation Buffer, vortexed for 5 s and incubated on ice for 5 min. After centrifuging for 5 min at 500× *g*, the supernatant is the cytoplasmic fraction. The pellet was resuspended in Membrane Isolation Buffer, vortexed for 15 s and incubated on ice for 5 min. After centrifuging for 5 min at 8000× *g*, the pellet was resuspended in Cytoskeleton/Nucleus Isolation Buffer, sonicated for 5 s at 20% power 3 times, and collected as the nuclear fraction.

### 4.11. Statistical Analysis

Data are presented as the mean ± SEM from at least three independent experiments. Normality of data distribution was assessed using the Shapiro–Wilk test. For comparisons between two groups, an unpaired two-tailed Student’s *t*-test was used for normally distributed data; otherwise, the Mann–Whitney U test was applied. For multiple-group comparisons, one-way ANOVA with Tukey’s post hoc test (parametric) or the Kruskal–Wallis test with Dunn’s post hoc test (non-parametric) was used. Two-way ANOVA was employed for comparisons involving two independent variables. All analyses were performed using GraphPad Prism 8.0.1, and a *p*-value < 0.05 was considered statistically significant.

## Data Availability

The original contributions presented in this study are included in the article. Further inquiries can be directed to the corresponding author.

## Abbreviations

The following abbreviations are used in this manuscript:

PD	Parkinson’s Disease
CMA	Chaperone-Mediated Autophagy
TFEB	Transcription Factor EB
CLEAR	Coordinated Lysosomal Expression and Regulation
CQ	Chloroquine

## Appendix B

**Table A1:** Primers designed for expression analysis in this study.

Gene Name	Primer Sequence (5′-3′)
*ATP6V0D2*-Forward	TCTGATCGAAACGCCATTAGC
*ATP6V0D2*-Reverse	CTTCTTTGCTCAATTCAGTGCC
*CTSD*-Forward	TGGACATCGCTTGCTGGAT
*CTSD*-Reverse	TTGGCTGCGATGAAGGTGAT
*CTSB*-Forward	GCCCGACCTACAAACA
*CTSB*-Reverse	GCCATTCTCCACTCCC
*CTSF*-Forward	ACAGAGGAGGAGTTCCGCACTA
*CTSF*-Reverse	GGGTCCCCTGGTTGAG
*LAMP1*-Forward	AGGACATACACTCACTCTC
*LAMP1*-Reverse	GTGCCACTAACACATCTG
*GLA*-Forward	TTCCTGCATCAGTGAGAAGCTC
*GLA*-Reverse	AGGTCTGGGCATCAATGTCG
*LC3*-Forward	CGCACCTTCGAACAAAGAGTAG
*LC3*-Reverse	AGATTGGTGTGGAGACGCTG
*SQSTM1*-Forward	ACTTGTGTAGCGTCTGCGAG
*SQSTM1*-Reverse	CACACTCTCCCCAACGTTCT
*ATG2A*-Forward	GTGCAAGTCAGCCTTCAAGC
*ATG2A*-Reverse	CTTCCCACCCTCATCCTTGG
*OPTN*-Forward	GATAGCTGGTGGTGCCACTT
*OPTN*-Reverse	TCCTGTGGAAAAGTCACTCCAAA
*GAPDH*-Forward	GAAGGTGAAGGTCGGAGTC
*GAPDH*-Reverse	TTGAGGTCAATGAAGGGG
